# Scorpion (Buthus martensii Karsch) in Chinese medicine: a review of traditional uses, chemical constituents, pharmacology, and toxicology

**DOI:** 10.3389/fphar.2025.1702650

**Published:** 2025-11-21

**Authors:** Meng-Han Liu, Juan-Juan Zhu, Qi Lu, Zhen-Ni Qu, Qiao Zhou, Lei Zhang, Yan-Peng Dai, Dian-Hua Shi

**Affiliations:** Shandong Academy of Chinese Medicine, Jinan, Shandong, China

**Keywords:** *Buthus martensii* Karsch, traditional uses, chemical constituents, pharmacological effects, toxicity

## Abstract

The processed *Buthus martensii* Karsch scorpions, commonly called Quanxie or *Scorpio,* have been a valuable traditional medicine for over a millennium, with documented use dating back to 935-960 AD. Traditionally employed in treating rheumatism, epilepsy, stroke, and chronic pain, *Scorpio* contains diverse chemical constituents, including venom, steroid derivatives, alkaloids, amino acids, and nucleosides. Modern pharmacological studies have identified active components, particularly venom and proteins, that exhibit analgesic, antitumor, antiepileptic, and antithrombotic properties. However, these same active ingredients can also induce neurotoxicity and autonomic dysfunction, with overdose leading to such adverse effects. Consequently, numerous processing methods have emerged to mitigate toxicity while preserving pharmacological activity. Despite these advances, key research gaps persist: modern studies overly rely on isolated venom components (with insufficient attention to multi-constituent interactions in processed whole scorpions), high-quality clinical trials are lacking in the functional research on active ingredients, and the mechanisms underlying processing-induced detoxification remain unclear.

## 1 Introduction

Processed scorpion *Buthus martensii* Karsch is a traditional animal-based medicine in Asia. The conventional processing method for scorpion medicinal material involves boiling live scorpions in salt water until their bodies become stiff, after which they are shade-dried (Commission, 2025). In this review, *Scorpio* refers to the dried body of the processed *B*. *martensii* scorpion. According to many ancient medical books in China, *Scorpio* has a medicinal history of more than 1,000 years (Zhang et al., 2024). The earliest record of *Scorpio* can be traced back to “*Shu Bencao”* (AD 935–960). Subsequent works, such as “*Kaibao Bencao”* (AD 973–974), “*Bencao Tujing”* (AD 1061), “*Compendium of Materia Medica”* (AD 1552–1,578) and “*Benjing Fengyuan”* (AD 1695), further recorded the medicinal properties (Zhang et al., 2024). There are 2,119 prescriptions containing *Scorpio* in the Dictionary of Traditional Chinese Medicine Formulas, and 34 kinds of preparations containing *Scorpio* are also included in the *Chinese Pharmacopoeia* (2025), such as *Dianxianping* tablets, *Kangshuan Zaizao* pills, and *Tongbi* capsules, which suggests a medicinal effect in treating stroke, epilepsy, rheumatism, hernia, chancre, and tetanus (Ma, 2019; Commission, 2025). South Korea, India, and countries in Africa also widely use *Scorpio* as a medicinal material (Yang et al., 2020). In modern medicine, *Scorpio* has also been used to treat gastric cancer, liver cancer, prostate cancer, and other tumors, as well as skin diseases and bronchial asthma (Shi and Guo, 2023), far exceeding the curative effects recorded in the Pharmacopoeia, which is closely related to advances in modern pharmacological research. Research indicates that *Scorpio* exhibits significant pharmacological effects including antitumor, antiepileptic, anticonvulsant, anticoagulant, and antibacterial properties (Wali et al., 2019). Additionally, they possess various biological activities such as analgesic and anti-inflammatory effects, growth promotion, and immune enhancement (Yang et al., 2020; Liu et al., 2021a). It is hypothesized that their pharmacological activities are primarily attributed to bioactive components like scorpion venom, steroidal derivatives, and alkaloids (Yang et al., 2020). Furthermore, in regions like Shandong and Henan Provinces, *Scorpio* is regarded as exceptional delicacy served at banquets, demonstrating high culinary value (Yang et al., 2020). This edible significance correlates with their rich nutritional composition containing amino acids, peptides, lipids, calcium, magnesium, and abundant trace elements (Song et al., 2024). It was found that *Scorpio* not only has obvious pharmacological effects, but also causes many adverse reactions such as abnormal liver function, cardiovascular system reactions, kidney injury, gastrointestinal reactions, and neurotoxic reactions (Yang et al., 2020). To mitigate its associated toxicity beyond dosage control, various processing methods–including boiling, mint-processing, licorice rinsing, and wine washing–are employed to reduce toxicity while enhancing therapeutic efficacy (Zhang et al., 2024). Nevertheless, the precise mechanisms underlying these effects remain poorly understood. At present, researchers have accumulated a large amount of research results on the chemical composition, pharmacological action, and toxicity of *Scorpio*. Based on this, this paper summarizes the above aspects in order to provide important reference materials for the in-depth application and development of *Scorpio*.

A comprehensive literature search covering the period from 1991 to 2024 was conducted using multiple electronic databases, including PubMed, Web of Science, Scienceing, Google Scholar, Elsevier, CNKI, WanFang, and WeiPu. The search terms included “*Buthus martensii* Karsch”, “Chinese Scorpion”, “Quanxie”, “traditional uses”, “chemical composition”, “pharmacology”, “toxicology”, and “processing”. In addition, Bopu Think Tank (https://www.bopuyun.com/) and Bolan Yishu (https://www.imedbooks.com/) provide information related to the Chinese Pharmacopoeia (2025 edition) and traditional Chinese medicine herbs. Studies with unclear experimental methods, insufficient sample sizes, or unvalidated outcome measures were excluded for quality filtering. Taxonomic identification of the studied scorpion species (*Buthus martensii* Karsch) was validated using the following authoritative resources: World Spider Catalog (integrated with Scorpion taxonomy, Version 2024 https://wsc.nmbe.ch/).

## 2 Traditional uses

The use of *Scorpio* in Chinese Traditional Medicine has a long history and is recorded in multiple classical Chinese medical texts. *Scorpio* was first documented as a medicinal substance in the *Shu Bencao*, although its efficacy and indications were not detailed therein (Ma, 2019). During the Song Dynasty, *Kaibao Bencao* began to document the therapeutic uses of *Scorpio* (Lu, 1995). Subsequent medical works in other historical periods successively added new records of its applications. It was not until the Qing Dynasty that a summary of its traditional uses was compiled, including dissipating blood heat, eliminating wind-dampness, resolving sores and ulcers, and serving as a channel-guiding agent (Ma, 2019). The traditional uses and initial documentation period of Scorpio are summarized in Table 1.

**TABLE 1:** Traditional uses and initial documentation period of *Scorpio*.

Period	Reference classics	Traditional uses	Modern pharmacological correlates
Song dynasty	*Kaibao Bencao* (Lu, 1995)	Stroke, hemiplegia, facial deviation (wry mouth and eyes)	Anticoagulant, Antithrombotic, Immunoregulation
		Spasms or convulsions	Anti-epilepsy, Anticonvulsant
		Wind-toxin induced urticaria/rashes (*engdu yinzhen*)	Antibiosis, Antiviral, Immunoregulation
	*Bencao Yanyi* (Kou, 1990)	Infantile convulsions (*jingfeng*)	Anticonvulsant
	*Baoqing Bencao Zhezhong* (Chen, 1991)	Resolve phlegm and dissipate nodules (*huatan sanjie*)	Antitumor, Immunoregulation
	*Gui Ren Fang* (Tang, 2011)	Deafness due to kidney deficiency	Immunoregulation
Jin and Yuan dynasty	*-* *-*	Hernial pain, rheumatic pain	Analgesic, Anti-inflammatory
		Gynecological disorders (such as leucorrhea)	Immunoregulation, Antibiosis
Ming dynasty	*Compendium of materia Medica* (Li, 1998)	External applications for hemorrhoids and ulcers	Anti-inflammatory, Antibiosis
		Localized treatments for disorders of the five senses and dermatology	Immunoregulation, Antibiosis

## 3 Chemical composition

The study of chemical constituents is the premise and foundation for clarifying the scientific connotation of traditional Chinese medicine in treating diseases (Li et al., 2023). The Chinese *Scorpio*, as an important animal-based traditional medicine, is very complicated in chemical composition. At present, many components such as protein peptides, steroid derivatives, alkaloids, fatty acids and amino acids have been found in scorpions.

### 3.1 Scorpion venom

Scorpion venom, secreted by *B*. *martensii* scorpion, can cause paralysis and even death. It has a complex composition, consisting of protein and non-protein components (Zhang et al., 2024). The protein portion is categorized into enzymes and peptides. To date, hyaluronidase, phospholipase A2, gelatinase, acetylcholinesterase, and nucleosidase have been found in scorpion venom (Yang et al., 2020). Peptides include protease inhibitors, bradykinin-potentiating peptides, histamine releasing factors, small molecular peptides, etc., among which small molecular peptides are also called scorpion toxins or neurotoxin peptides (Zhang et al., 2024). Scorpion venom toxins consist of 20–80 amino acids, contain 3-4 pairs of disulfide bonds, and have molecular weights ranging from 6,000 to 9,000 Da (Yang et al., 2020). These toxins can exert pharmacological effects by acting on corresponding receptor sites or ion channels (K^+^, Ca^2+^, Na^+^, Cl^−^), and are the main active components responsible for *Scorpio*’s pharmacological effects (Chen et al., 2001; Zheng et al., 2024).

According to the bond connections, scorpion toxins can be divided into disulfide-bridged peptides (DBPs) and non-disulfide-bridged peptides (NDBPs) (Almaaytah and Albalas, 2014; Rincón-Cortés et al., 2019). The main function of disulfide bonds in DBPs is to maintain the stability of scorpion toxins and exhibit various neurotoxic and cytotoxic effects (Zhang and Wang, 2007). With the in-depth study, it has been found that they can specifically act on ion channels (Na^+^, K^+^, Ca^2+^, Cl^−^) on the membrane to play antibacterial, anticancer, anti-inflammatory, and immunomodulatory roles (Cyril et al., 2002; Almaaytah and Albalas, 2014). NDBPs have pharmacological effects such as antibacterial, bradykinin enhancement, hemolysis, and immunomodulation (Vega et al., 2010). Information on the types and pharmacological effects of scorpion venom peptides is shown in Table 2.

**TABLE 2:** Scorpion venom peptides contained in *B. martensii* scorpion and their pharmacological action.

Types of scorpion venom peptide	Polypeptide name	Sequences	Pharmacological action	References
Targeted Na^+^ channels	CvIV4	MNYFILILVAALLILDVNCKKDGYPVEHSGCKYTCWKNEYCDK VCKDLKGEGGYCYINLTCWCTGLPDNVPLKTNQRCNGKRK	Analgesic	Yang et al. (2020)
	MKTxⅢ	MNYLIVISFALLLMTGVESGRDAYIAKKENCTYFCALNPYCND LCTKNGAKSGYCQWAGRYGNACWCIDLPDKVPIRIPGPCIGR	Analgesic	Cyril et al. (2002)
	BmK Ⅰ1	-	Analgesic	Cyril et al. (2002)
	BmK Ⅰ4	-	Analgesic	Shao et al. (2007a)
	BmK Ⅰ6	-	Analgesic	Shao et al. (2007a)
	BmK I	VRDAYIAKPHNCVYECARNEYCNDLCTKNGAKSGYCQW VGKYGNGCWCIELPDNNVPIRVPGKCH	Pro-epilepsy	Xiao et al. (2022)
	BmK IT1	KKNGYAVDSSGKVSECLLNNYCNNICTKVYYATSGYCC LLSCYCFGLDDDKAVLKIKDATKSYCDVQIIG	Anti-epilepsy and Anticonvulsant	Zhao et al. (2008)
	BmK IT2	DGYIKGKSGCRVACLIGNQGCLKDCRAYGASYGYCW TWGLACWCEGLPDNKTWKSESNTCG	Anti-epilepsy and Anticonvulsant	Zhao et al. (2008)
	BmK IT-AP	KKNGYAVDSSGKVAECLFNNYCNNECTKVYYAD KGYCCLLKCYCFGLADDKPVLDIWDSTKNYCDVQIIDLS	Analgesic	Shao et al. (2007a)
	BmK M10	MNYLVMISFALLLMKGVESVRDAYIAKPENCVYECGITQD CNKLCTENGAESGYCQWGGKYGNACWCIKLPDSVPIRVPGKCQR	Antithrombotic	Cyril et al. (2002)
	MKTxI	GRDAYIADSENCTYTCALNPYCNDLCTKNGAKSGY CQWAGRYGNACWCIDLPDKVPIRISGSCR	Nitrate energy reaction	Cyril et al. (2002)
	Bukatoxin	VRDGYIADDKNCAYFCGRNAYCDEECIINGAESGYC QQAGVYGNACWCYKLPDKVPIRVSGECQQ	Analgesic	Cyril et al. (2002)
	Css4	MNSLLMITACLALVGTVWAKEGYLVNSYTGCKFECFKLGDNDYCL RECRQQYGKGSGGYCYAFGCWCTHLYEQAVVWPLPNKTCNGK	Analgesic	Liu et al. (2016)
	BmK AngP1	KKNGYAVDSSGKVAE	Analgesic	Shao et al. (2007a)
	BmK Ang M1	MNYLVMISFA LLLMKGVESV RDAYIAKPEN CVYECGITQD CNKLCTENGA ESGYCQWGGK YGNACWCIKLPDSVPIRVPGKCQR	Analgesic, Anti-Inflammatory	Shao et al. (2007a)
	BmK IT3	DGYIRGSNGCKISCLWGNEGCNKECKGFGAYYGYCWTW GLACWCBGLPDDKTWKSESNTCG	Kill insects	Cyril et al. (2002)
	BmK IT4	DGYIRGSNGCKISCLWGNEGCNKECKGFGAYYGY CWTWGLACWCBGLPDDKTWKSESNTCG	Kill insects	Cyril et al. (2002)
	BmK IT5	DGYIKRHDGCKVTCLINDNYCDTECKREGGSYG YCYSVGFACWCEGLPDDKAWKSETNTCD	Kill insects	Cyril et al. (2002)
	BmK d IT-AP3	-	Analgesic, Anti-Epilepsy and Anticonvulsant	Shao et al. (2007a)
	BmK AS1	DNGYLLDKYTGCKIWCVINNESCNSECKLRRGNYGYC YFWKLACYCEGAPKSELWAYETNKCNKGM	Analgesic, Anti-Inflammatory	Cyril et al. (2002)
	BmK AS	DNGYLLDKYTGCKVWCVINNESCNSECKIRGGYY GYCYFWKLACFCQGARKSELWNYNTNKCNGKL	Anti-inflammatory, Analgesic, and Anticonvulsant	Zhao et al. (2011a), Liu et al. (2012)
	BmK ANEP	MKLSLLLVISASMLIDGLVNADGYIRGSNGCKVSCLWGNDGCNKEC RAYGASYGYCWTWGLACWCEGLPDDKTWKSESNTCGGKK	Analgesic	Lu et al. (2022), Song et al. (2011)
	BmK AGAP	VRDGYIADDKNCAYFCGRNAYCDDECKKNGAES GYCQWAGVYGNACWCYKLPDKVPIRVPGKCNGG	Analgesic, Antitumor	Zhao et al., 2011b; Liu (2013), Li et al. (2016), Xu et al. (2017)
	BmNal-3SS2	-	Analgesic	Cui et al. (2017)
	BmK bpp	FRFGSFLKKVWKSKLAKKLRSKGKQLLKDYANKVLNGPEEEAAAPAE	Antibiosis	Cyril et al. (2002)
	BmK BTx	MMKLVLFGIIVILFSLIGSIHGISGNYPLNPYGGYYYCTILGENEYCK KICRIHGVRYGYCYDSACWCETLKDEDVSVWNAVKKHCKNPYL	Analgesic	Cui et al. (2017)
	BmK AEP	DGYIRGSNGCKVSCLLGNEGCNKECRAYGASYGY CWTYKLACWCBGLPDDKTWKSESNTCG	Anti-epilepsy	Wang et al. (2010a)
	BmK *α*IV	MNYLVFFSLALLLMTGVESVRDGYIADDKNCAYFCGRNAYCDDEC KKKGAESGYCQWAGVYGNACWCYKLPDKVPIRVPGRCNGG	Pro-epilepsy	Xiao et al. (2022)
	BmK NT1	-	Neurotoxicity	He et al. (2017)
	BmK M2	VRDAYIAKPHNCVYECARNEYCNNLCTKNGAKS GYCQWSGKYGNGCWCIELPDNVPIRVPGKCH	Neurotoxicity	He et al. (2000)
	BmK M7	VRDGYIALPHNCAYGCLNNEYCNNLCTKDGAKI GYCNIVGKYGNACWCIQLPDNVPIRVPGRCHPA	Cardiotoxicity	Guan et al. (2002)
Targeted K^+^ Channels	BmKTX	VGINVKCKHSGQCLKPCKDAGMRFGKCINGKCDCTPK	Analgesic, Immunoregulation	Yang et al. (2020)
	BmKKx2	RPTDIKCSASYQCFPVCKSRFGKTNGRCVNGLCDCF	Antitumor	Jian et al. (2013)
	BmKTT-1	QKDCSLPVDTGRGKGWFLRYYYNKNSKTCES FIYGGVGGNKNNFLNIENCCKICKAKNC	Inhibit protease activity	Chen et al. (2012)
	BmKTT-2	VDCTLPSDTGRCKAYFIRYFYNQKAGECQ KFVYGGCEGNSNNFLTKSDCCKQCSPGKC	Inhibit protease activity	Chen et al. (2012)
	BmKTT-3	SINCRLPPERGPCRGNITKYYYHNESRTCRT FSYGGCEGNSNNFRNRHYCMKYCARKRH	Inhibit protease activity	Chen et al. (2012)
	BmTX1	MKISFLLLALVICSIGWSEAQFTDVKCTGS KQCWPVCKQMFGKPNGKCMNGKCRCYS	Kill insects	Cyril et al. (2002)
	BmTX2	MKISFLLLLAIVICSIGWTEAQFTNVSCSAS SQCWPVCKKLFGTYRGKCMNSKCRCYS	-	Cyril et al. (2002)
	BmTX3B	MKIFSILLVALIICSISICTEAFGLIDVKCFAS SECWTACKKVTGSGQGKCQNNQCRCY	-	Cyril et al. (2002)
	BmTXK-*β*	MMKQQFFLFLAVIVMISSVIEAGRGKEIMKNIKEKLTEVKDKMKHS WNKLTSMSEYACPVIEKWCEDHCAAKKAIGKCEDTECKCLKLRK	Antibiosis	Almeida et al. (2013)
	BmP01	ATCEDCPEHCATQNARAKCDNDKCVCEPK	-	Cyril et al. (2002)
	BmP02	VGCEECPMHCKGKNAKPTCDDGVCNCNV	-	Cyril et al. (2002)
	BmP03	VGCEECPMHCKGKNANPTCDDGVCNCNV	-	Cyril et al. (2002)
	BmP05	AVCNLKRCQLSCRSLGLLGKCIGDKCECVKH	-	Cyril et al. (2002)
	IbTX	QFTDVDCSVSKECWSVCKDLFGVDRGKCMGKKCRCYQ	Anti-inflammatory	Yang et al. (2020)
	BmSKTx1	MKIFFAILLILAVCSMAIWTVNGTPFAIKCA TNADCSRKCPGNPPCRNGFCACT	-	Cyril et al. (2002)
	LmKTT	MKISFVLLLTLFICSIGWSEARPTDIKCSASYQ CFPVCKSRFGKTNGRCVNGLCDCF	Immunoregulation	Yang et al. (2020)
	Kbot21	AACYSSDCRVKCRAMGFSSGKCIDSKCKCYK	Immunoregulation	Yang et al. (2020)
	Anuroctoxin	QKECTGPQHCTNFCRKNKCTHGKCMNRKCKCFNCK	Immunoregulation	Yang et al. (2020)
	Urotoxin	MNAKLIYLLLVVTTMMLTFDTTQAGDIKC SGTRQCWGPCKKQTTCTNSKCMNGKCKCYGCVG	Immunoregulation	Yang et al. (2020)
	Maurotoxin	VSCTGSKDCYAPCRKQTGCPNAKCINKSCKCYGC	Immunoregulation	Yang et al. (2020)
	HeTx204	GTVYVFLLLLAFGIFTDISNACSEQMDDEDSYE VEKRGNACIEVCLQHTGNPAECDKPCDK	Immunoregulation	Yang et al. (2020)
	OcyKTx2	IRCQGSNQCYGHCREKTGCMNGKCINRVCKCYGC	Immunoregulation	Yang et al. (2020)
	ImKTx10	-	Immunoregulation	Zhang et al. (2022b)
	ImKTx58	-	Immunoregulation	Zhang et al. (2022b)
	ImKTx88	-	Immunoregulation	Zhang et al. (2022b)
	ImKTx104	MNFWVTFIRLIVVLSIVFAFQIAVAKA ACVTHEDCTLLCYDTIGTCVDGKCKCM	Immunoregulation	Yang et al. (2020)
	ADWX-1	VGINVKCKHSRQCLKPCKDAGMRFGKCTNGKCHCTPX	Immunosuppression	Cyril et al. (2002)
	BmTxKS1	MNRLTTIILMLIVINVIMDDISESKVAAGIVCK VCKIICGMQGKKVNICKAPIKCKCKKG	-	Cyril et al. (2002)
	BmTxKS2	MTYAILIIVSLLLISDRISNVVDKYCSENPLD CNEHCLKTKNQIGICHGANGNEKCSCMES	Antibiosis	Cyril et al. (2002)
	CoTX1	MEGIAKITLILLFLFVTMHTFANWNTEAAVCVY RTCDKDCKRRGYRSGKCINNACKCYPYGK	Neurotoxicity	Yang et al. (2020)
	Agitoxin1	GVPINVKCTGSPQCLKPCKDAGMRFGKCINGKCHCTPK	Neural regulation	Yang et al. (2020)
	Agitoxin2	GVPINVSCTGSPQCIKPCKDAGMRFGKCMNRKCHCTPK	Neural regulation	Yang et al. (2020)
Targeted Ca^2+^ Channels	Kurtoxin	KIDGYPVDYWNCKRICWYNNKYCNDLCKGLK ADSGYCWGWTLSCYCQGLPDNARIKRSGRCRA	Neural regulation	Yang et al. (2020)
	Martentoxin	MKIFSILLVALIICSISICTEAFGLIDVKCFAS SECWTACKKVTGSGQGKCQNNQCRCY	Neural regulation	Yang et al. (2020)
	BmK-YA	MIFHQFYSILILCLIFPNQVVQSDKERQDWIPSDYGGYMNPAGRSD EERQDWIPSDYGGHMNPAGRSDEERQDWIPSDYGGHMNPAGRSN EERQDWIPSDYGGYMNPAGRSDEERQDWIPSDYGGHMNPAGRSN EERQDWIPSDYGGYMNPAGRSDEERQDWIPSDYGGHMNPAGRSD EERQDWIPSDYGGYMNPAGRSD	Analgesic	Yang et al. (2020)
Targeted Cl^−^ Channels	Bm-12	CGPCFTTDANMARKCRECCGGIGKCFGPQCLCNRT	-	Wu et al. (2000)
	Chlorotoxin	MKFLYGIVFIALFLTVMFATQTDGCGPCFTTDANMA RKCRECCGGIGKCFGPQCLCNRI	Cell proliferation promotion	Yang et al. (2020)
	Bs-Tx7	-	Cell proliferation promotion	Yang et al. (2020)
	BmK AGP-SYPU1	GRDAYIAQNYNCVYHCFRDDYCNGLCTENGADS GYCYLAGKYGHACWCINLPDDKPIRIPGKCHRR	Analgesic	Meng et al. (2015)
	BmK AGP-SYPU2	MNYMVIISLALLVMTGVESVKDGYIADDRNCPYFCGRNAYCDGEC KKNRAESGYCQWASKYGNACWCYKLPDDARIMKPGRCNGG	Cell proliferation promotion Analgesic	Zhang et al. (2011), Yang et al. (2020)
	BmKn2	FIGAIARLLSKIF	Anti-cancer, Antibiosis	Cao et al. (2012), Satitmanwiwat et al. (2016)
	BmK CT	CGPCFTTDANMARKCRECCGGIGKCFGPQCLCNRI	Anti-cancer	Li et al. (2019)

Protein peptides in scorpion venom, as the main chemical components, have been widely studied at present. Transcriptomic and proteomic analyses have been applied to detect the potential toxins of scorpions from different habitats and instars (Gao et al., 2021; Guo et al., 2024). However, it is still unknown whether these substances remain in *Scorpio* used as traditional Chinese medicine materials. Proteomic analysis was used to comprehensively characterize the functional toxins in *Scorpio*. A total of 66 heat-resistant potassium channel-regulating toxins were identified, and various degraded toxin fragments were also detected, indicating that their relative thermal instability may reduce toxicity (Yang et al., 2019).

### 3.2 Steroid derivatives

Steroidal compounds are widely distributed in nature. Currently, 6 steroidal compounds have been isolated from *B. martensii*, including cardenolides and cholesterol derivatives. Pharmacological activity studies have shown that cardenolides exhibit antibacterial, cardiotonic, and antitumor activities, among others; cholesterol derivatives also possess activities such as antibacterial, antitumor, antiviral, and anti-inflammatory effects (Gao, 2012; Gao et al., 2014). These steroidal compounds are hypothesized to be the material basis for the pharmacological activities of *Scorpio*, such as its antitumor and anti-inflammatory effects.

#### 3.2.1 Cardiac glycosides

**FIGURE 1:**
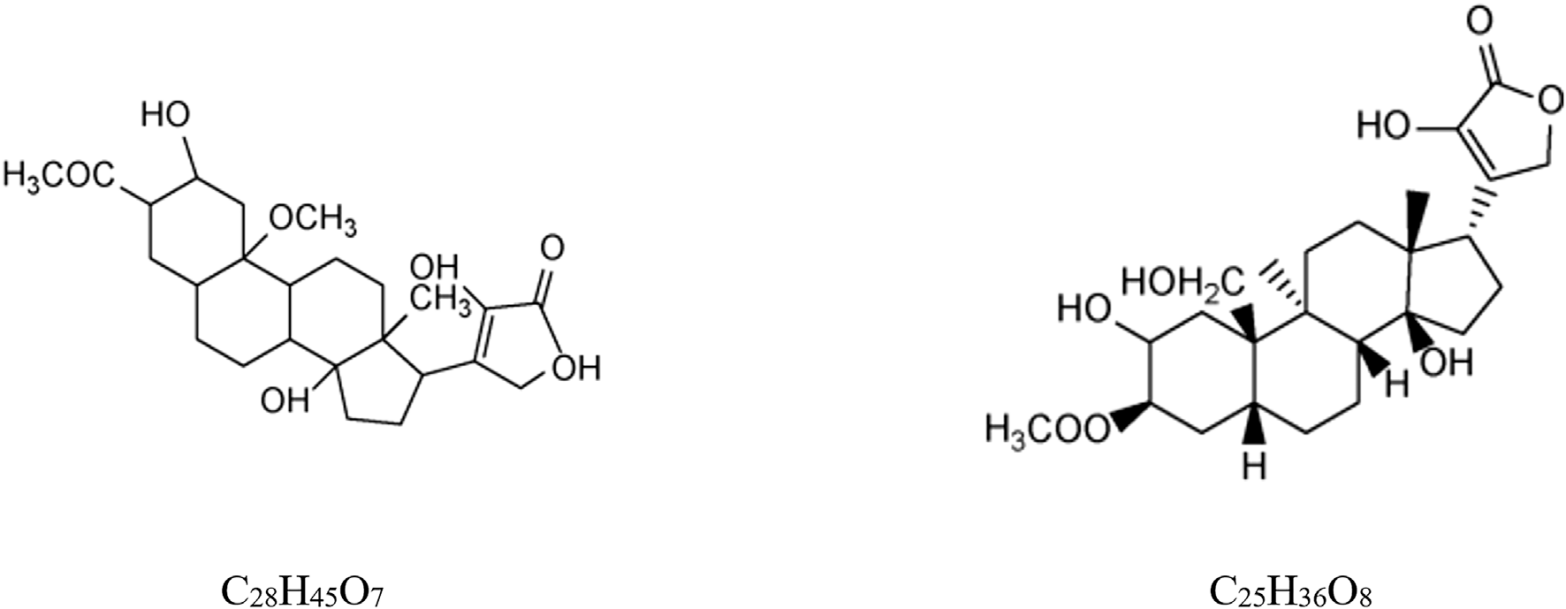
Structures of cardiac glycosides from *B. martensii*

To date, two specific cardiac glycosides with lactone alcohol structures have been identified from *Scorpio*: 2*β*-,22-dihydroxy,3-acetoxyl,20-methoxy-cardenolidol (C_28_H_45_O_7_) (Gao, 2012) and 3*β*-acetoxyl,2,14,22-trihydroxy,19-hydroxymethyl,9a,5*β*,14*β*-card-20 (22)enolide (C_25_H_36_O_8_) (showed in Figure 1) (Gao et al., 2014).

#### 3.2.2 Cholesterol and its analogues

**FIGURE 2:**
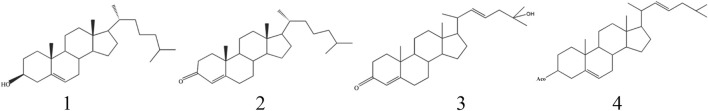
Structures of cholesterol components from *B. martensii*.

The initial identification of cholesterol in *B. martensii* scorpion was reported in 2012 (Gao, 2012). Subsequent phytochemical investigations have led to the isolation and characterization of four distinct sterol compounds from *B. martensii*, whose detailed chemical structures are systematically presented in Table 3 and Figure 2.

**TABLE 3:** Cholesterol components in *B. martensii*.

No.	Compound	Molecular formula	Molecular weight	Reference
1	Cholesterol	C_27_H_46_O	386.7	Gao (2012)
2	Cholest-4-en-3-one	C_27_H_44_O	383.0	Ai (2017)
3	(−)22E,24-3-cholestone-4,22 (23)-diene-25-alcohol	C_27_H_43_O_2_	427.0	Liu (2018)
4	(−)5(6),22(23)-Cholestadien-3*β*-acetoxy ester	C_29_H_47_O_2_	494.0	Liu (2018)

### 3.3 Alkaloids

**FIGURE 3:**
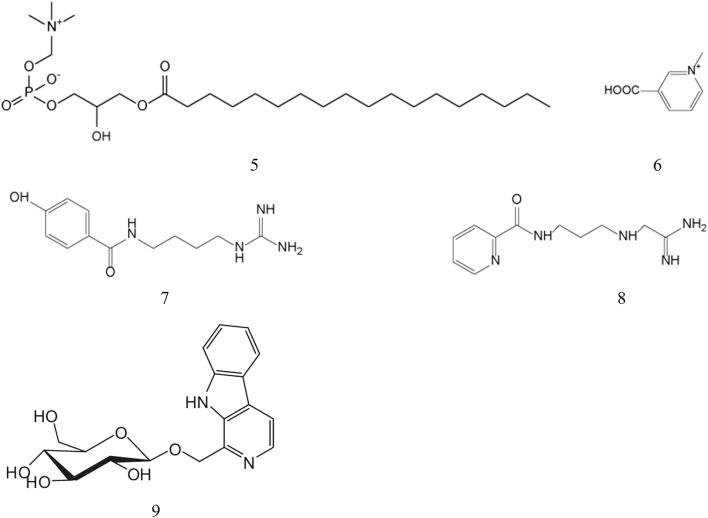
Structures of alkaloids from *B. martensii*.

Alkaloids represent a diverse class of naturally occurring nitrogen-containing organic compounds with significant pharmacological potential. Extensive phytochemical investigations have identified several alkaloids in *B. martensii*, including 1-stearyl- glycerol-3-phosphorylcholine, trigonelline, martensine A and martensine B, whose structural details are systematically presented in Table 4 and Figure 3.

**TABLE 4:** Alkaloid components in *B. martensii*.

No.	Compound	Molecular formula	Molecular weight	Reference
5	1-stearyl- glycerol-3-phosphorylcholine	C_26_H_55_NO_7_P	524.4	Ai (2017)
6	trigonelline	C_7_H_8_NO_2_	138.0	Ai (2017)
7	martensine A	C_12_H_18_N_4_O_2_	250.2	Fan (2020)
8	martensine B	C_11_H_18_N_5_O	236.2	Fan (2020)
9	harmanyl-*β*-D-glucopyranoside	C_18_H_21_ N_2_O_6_	361.4	Kim (2013)

Pharmacological studies have demonstrated that scorpion-derived alkaloids exhibit a broad spectrum of bioactivities, including antimicrobial, antitumor, anti-aging, hypoglycemic, hypolipidemic, neuroprotective, and cognitive-enhancing properties (Kamble and Bodhankar, 2013; Pravalika et al., 2018; Zeng et al., 2021). Notably, martensine A and martensine B have shown remarkable propidium iodide substitution capacity, suggesting their potential therapeutic application in Alzheimer’s disease treatment (Fan, 2020). Furthermore, the isolation of harmanyl-*β*-D-glucopyranoside, a novel *β*-carboline glucopyranoside from *B. martensii*, has revealed significant hypoglycemic activity, highlighting the therapeutic potential of scorpion alkaloids in diabetes management.

### 3.4 Odor components

The characteristic fishy odor of *Scorpio* is primarily attributed to their volatile organic compounds, including aldehydes, terpenoids, and aromatic compounds with distinct olfactory properties (Yang et al., 2020).

**FIGURE 4:**
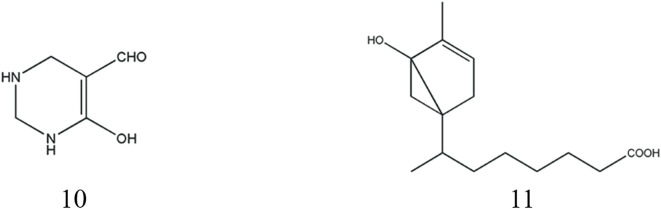
Structures of Odor components from *Scorpio*.

In 2012, a groundbreaking discovery revealed the presence of a unique nitro aldehyde structure in scorpions, specifically identified as 6-hydroxy-1-ene-tetrahydro-pyrimidine aldehyde, representing a hydroxyl-containing alkanal (Gao, 2012). Terpenoids, characterized by their structural composition of two or more isoprene units, were further investigated in scorpions. Notably (−)4-(2′-iso-octanoic acid)-6-hydroxy-1-methyl cyclohexene, a carboxyl-containing terpene, was first isolated from scorpions in 2018 (Liu, 2018). The structural details are presented in Table 5; Figure 4. Through advanced analytical techniques, researchers have employed headspace solid-phase microextraction (HS-SPME) coupled with gas chromatography-mass spectrometry (GC-MS) to comprehensively profile the volatile components of scorpions. A total of 43 components were separated and 42 components were identified, including aldehydes and ketones (11 species), terpenoids (8 species), and aromatics (4 species). The main chemical constituents are benzaldehyde (19.03%), phenol (13.22%), 2-pentylfuran (7.42%) and cubeba olefine (7.31%) (Zhou et al., 2018). In order to remove the fishy smell, doctors in past dynasties fried scorpion and glutinous rice together or prepared them with vinegar to absorb and mask the unpleasant odor-causing substances (Du et al., 2024).

**TABLE 5:** Odor components in *Scorpio*.

No.	Compound	Molecular formula	Molecular weight	Reference
10	6-hydroxy-1-ene-tetrahydro-pyrimidine aldehyde	C_5_H_8_N_2_O_2_	129	Gao (2012)
11	(−)4-(2′-iso-octanoic acid)-6-hydroxy-1-methyl cyclohexene	C_15_H_25_O_3_	253	Liu (2018)

### 3.5 Nutritional ingredients

*Scorpio* represents a valuable source of essential nutrients, comprising amino acids, lipids, and mineral elements that are crucial for maintaining human physiological functions and promoting overall health (Yang et al., 2020).

#### 3.5.1 Amino acids

As fundamental building blocks of proteins, amino acids play a pivotal role in sustaining vital biological processes. Extensive biochemical analyses have revealed that scorpions contain a diverse array of amino acids, with more than 17 distinct types identified, including 6 essential amino acids (Li et al., 2007). Notably, the amino acid profile of scorpions exhibits significant variation depending on geographical origin and processing methodologies. This variability in both quantitative composition and qualitative distribution of amino acids has been established as a reliable biochemical marker for assessing the quality of scorpion-derived products (Li et al., 2007; Liang et al., 2014).

#### 3.5.2 Lipids

Lipids are essential substances for the human body to store and supply energy, including lipids (phospholipids) and oils. The phospholipid composition of *Scorpio* has been extensively characterized, with particular attention to lecithin components that feature hydrophilic phosphorylcholine groups (Ai, 2017). Specifically, scorpions contain a unique phospholipid identified as 1-stearyl-glycero-3-phosphocholine, characterized by its long-chain fatty acyl group structure. In addition, scorpion oil contains many unsaturated fatty acids such as palmitic acid, stearic acid, oleic acid, linoleic acid, linolenic acid, and behenic acid (Uchiyama et al., 2010; Ferreira et al., 2015).

#### 3.5.3 Other ingredients

Nucleosides represent fundamental biomolecules that play crucial roles in cellular metabolism and the maintenance of vital biological processes. *Scorpio* has been found to contain a diverse array of nucleosides, including uracil, hypoxanthine, xanthine, guanosine, and adenosine (Zhang et al., 2024). Notably, the concentration profile of these nucleoside components exhibits significant variation depending on the extraction methodology employed, with different decoction techniques resulting in markedly distinct nucleoside compositions in the final extract (Tian et al., 2013). In addition to nucleosides, scorpions are a rich source of essential minerals, particularly calcium (Ca) and magnesium (Mg), along with various trace elements that are vital for human physiological functions. These inorganic nutrients are predominantly concentrated in the abdominal region of scorpions (Ma and Lin, 2000). Interestingly, comparative analyses have revealed that female scorpions contain higher concentrations of Ca and Mg compared to their male counterparts. This sexual dimorphism in mineral content has been proposed as a potential biomarker for quality assessment in the evaluation of premium-grade scorpion specimens (Ma and Lin, 2000).

Functional active components in scorpions have been described, but how many effective substances are retained in processed scorpion medicinal materials? Integrated transcriptomics–proteomics–metabolomics analysis can be used to track the formation patterns of degraded scorpion venom peptide fragments and other small molecules after processing, thereby enriching the research on the material basis of *Scorpio*.

## 4 Pharmacological effects

**FIGURE 5:**
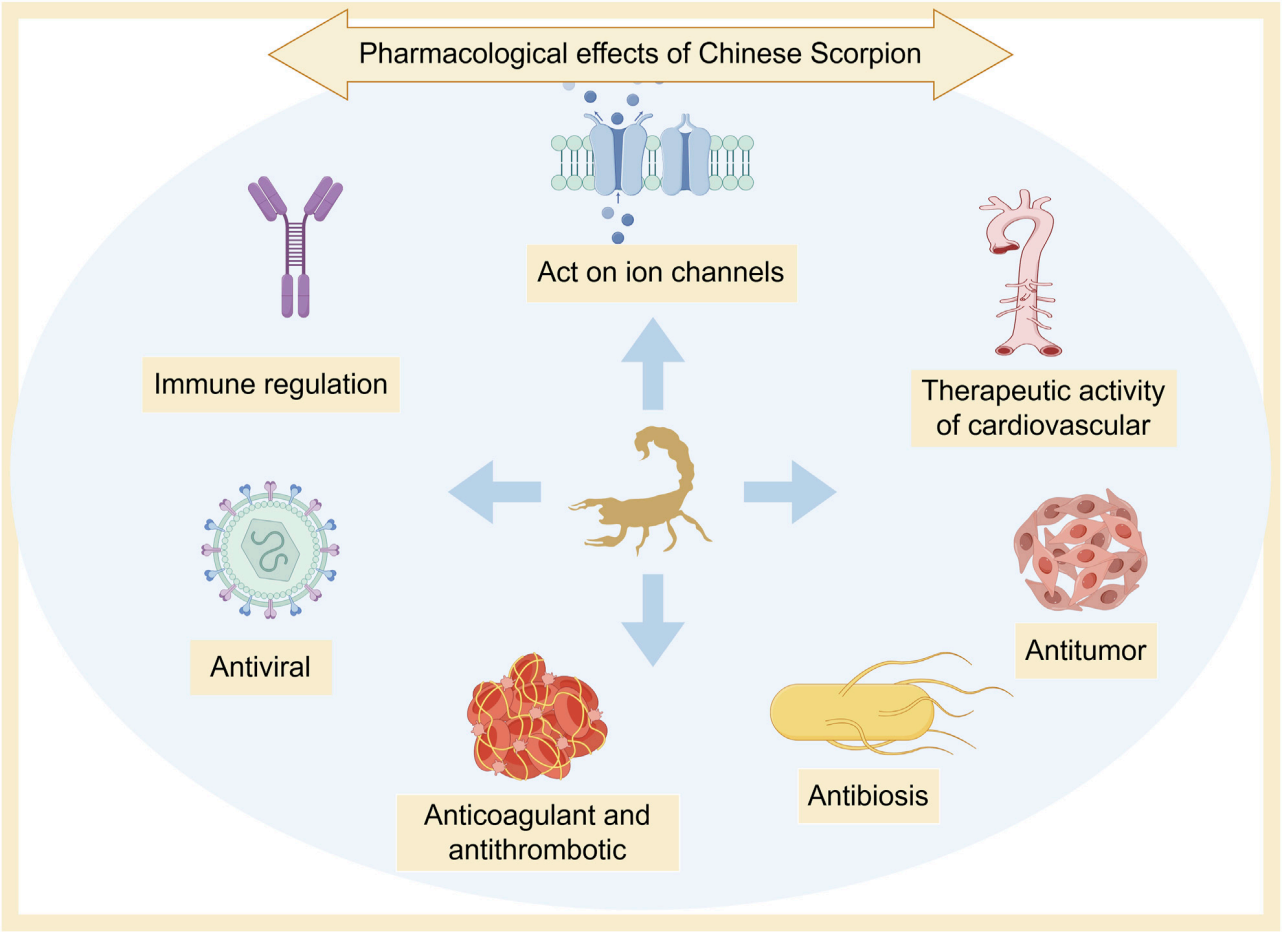
Pharmacological effects of *Scorpio*.

*Scorpio* has a long history of medicinal use. It is an important medicinal material for treating spasms and convulsions, and has the functions of detoxifying and dredging collaterals (Ma, 2019). Modern research shows that *Scorpio* has many pharmacological activities, such as anti-epilepsy, anticonvulsant, anti-tumor, bacteriostatic, anticoagulant, and antithrombotic effects (Wali et al., 2019; Yang et al., 2020) (Figure 5).

### 4.1 Anticancer activity

**FIGURE 6:**
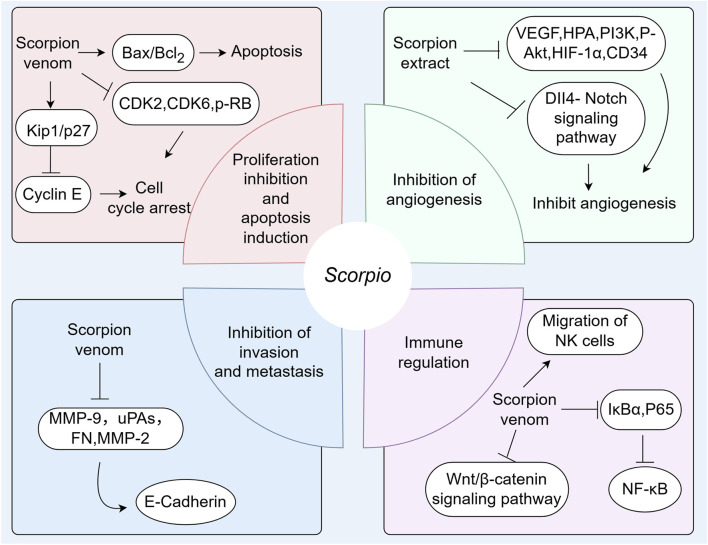
Mechanisms of *Scorpio*’s anti-tumor activity.

Although great progress has been made in cancer treatment, the morbidity and mortality are still increasing. Therefore, it is still urgent to develop new and effective anticancer drugs. The crude peptide extracts and peptide monomers from the venom of *B*. *martensii* scorpions show anti-cancer effects, which indicates that the venom is a good source for finding new anti-cancer drugs (Wang and Ji, 2005). A large number of studies have confirmed that scorpion venom polypeptides have shown good effects in the treatment of liver cancer, lung cancer, glioma, brain tumor, breast cancer, melanoma, prostate cancer and esophageal cancer (Fu et al., 2007; Mishal et al., 2013). The mechanisms of anti-tumor activity are summarized in Table 6 and Figure 6.

**TABLE 6:** The anti-tumor activity of *B*. *martensii* scorpions.

Name	Effective concentration	Cellular/Animal model	Mode of action	Reference
PESV	50–100 mg/L	Human bladder carcinoma T24 cells	Upregulate pro-apoptosis protein Bax and downregulate anti-apoptosis protein Bcl-2	Hou et al. (2013)
	40 mg/kg	Lewis lung carcinomas	Reduce the expression of DII4 and Notch1	Sun et al. (2011)
	10–40 mg/L	Pancreatic cancer MIA-paca-2 cells	Inhibit the secretion of MMP-9 and upa, reduce FN levels, upregulate the expression of E-Cadherin protein	Zhang et al. (2009)
	10 mg/kg, 40 mg/kg	H22 Hepatocellular Carcinoma Subcutaneously Implanted Tumor Model	Increase the expression of PTEN and decrease the expression of PI3K、P-Akt、COX-2、HIF-1*α* and VEGF-A	Sui et al. (2014)
	10 mg/kg, 40 mg/kg	H22 Hepatocellular Carcinoma Subcutaneously Implanted Tumor Model	Increase the proportion of NK cells infiltrating tumor tissues, restore NK cell cytotoxic activity	Han et al. (2016)
	25–200 mg/L	Non-small cell lung cancer cell line A549 cells	Increase the expression of PTEN and decrease the expression of HIF-1*α* and VEGF, arrest the cell cycle in the G_0_/G_1_ phase	Wang et al. (2012)
	10 mg/kg, 20 mg/kg	Human SKOV3 Nude Mouse Xenograft Tumor Model	Inhibit the expression of PI3 K and P-Akt and increase PTEN in the microenvironment of tumors	Song et al. (2012b)
SVCIII	29 μg/mL and 39.6 μg/mL	Human leukemia THP-1cells and Jurkat cells	Induce cell cycle arrest in the G_1_ phase and inhibit the activation of NF-κB	Song et al. (2012a)
rBmk AGAP	10–30 μM	Human gliomas SHG-44 cells	Inhibit the expression of cell cycle regulatory proteins CDK2, CDK6, and p-RB, arrest the cell cycle in the G_1_ phase, and interfere with the p-AKT, NF-kb, BCL-2, and MAPK signaling pathways	Zhao et al. (2011b)
	15–60 μM	Human breast cancer MDA-MB-231 and MCF-7 cells	Decrease the expression of Oct4, Sox2, N-cadherin, Snail, and increase the expression of E-cadherin.	KAMPO (2019)
	1.0 mg/kg	S-180 fibrosarcoma model	Inhibit the growth of solid tumors and prolong the survival days	Liu et al. (2003)
rBmk CTa	0.28 μM	Human glioma SHG-44 cells	Inhibit the proliferation	Fu et al. (2007)
Bmk CT	0.6–2.4 μM	Rat glioma C6 cells	Inhibit the invasion and migration by antagonizing MMP-2	Fu et al. (2011)

It has been found that scorpion venom polypeptides can inhibit the proliferation of many cancer cell lines. The main mechanisms are to induce cancer cell apoptosis, cell cycle arrest, inhibition of angiogenesis, invasion and migration, and immune regulation. Vascular endothelial growth factor (VEGF), epidermal growth factor receptor (EGFR), and basic fibroblast growth factor (bFGF) are highly expressed in various tumors and are considered important signal transducers in angiogenesis, involving the MAPK (Mitogen-Activated Protein Kinase), PI3K (Phosphoinositide 3-kinase)/Akt, and other signaling pathways (Sui et al., 2014). Many research results suggest that scorpion extract can inhibit the expression of VEGF, HPA, PI3K, P-Akt, HIF-1*α*, and CD34 in the microenvironment of liver cancer, thereby inhibiting angiogenesis (Wang et al., 2010b; Sui et al., 2014). The invasion and metastasis of tumor cells constitute one of the primary factors contributing to disease progression and deterioration. Currently, numerous studies focus on processes such as epithelial-mesenchymal transition (EMT) and the matrix metalloproteinase family (MMPs). Following EMT, epithelial cells undergo a series of cellular transformations ultimately resulting in basement membrane degradation and extracellular matrix remodeling (Patrick Henriet and Hervé Emonard, 2019). The MMP family is extensive and capable of degrading various types of collagen and adhesion proteins, thereby playing a crucial role in tumor cell adhesion, invasion, and migration (Patrick Henriet and Hervé Emonard, 2019). Tumor cells evade immune recognition and attack through complex mechanisms, allowing unchecked growth. Blocking this immune escape and remobilizing the immune system is a key anti-tumor strategy (Uzhachenko et al., 2025).

PESV (polypeptide extract from scorpion venom) is a group of 50–60 amino acids long peptides extracted from *B*. *martensii* crude venom. And it exhibits inhibitory effects on a variety of cancer cells. In addition to inducing tumor cell apoptosis, arresting the cell cycle, and inhibiting angiogenesis, PESV significantly suppress liver metastasis of pancreatic cancer with an inhibition rate exceeding 70%, which is markedly superior to the anti-angiogenic drug TNP-470 (Cui et al., 2009; Zhang et al., 2009). BmK AGAP is a long-chain toxin isolated from *B*. *martensii* venom, which contains 66 amino acid residues and 4 pairs of disulfide bridges (Zeng et al., 2000; Liu et al., 2002). Voltage-gated Na^+^ ion channel Nav1.5 is overexpressed in breast cancer and is associated with tumor progression (Brackenbury, 2012). BmK AGAP downregulates PTX3 expression by inhibiting or binding to Nav1.5, thereby reducing the activation of the NF-*κ*B and Wnt/*β*-catenin signaling pathways as well as the production of the inflammatory factor TNF-*α*(Kampo et al., 2019). This demonstrates the potential of BmK AGAP in the treatment of inflammation-induced cancers. Recombinant BmK AGAP is more effective for tumor cells and less harmful for healthy cells than cyclophosphamide in S-180 fibrosarcoma model (Liu et al., 2003). In summary, both natural polypeptides and recombinant polypeptides exhibit great potential in antitumor applications. Meanwhile, the high expression efficiency and high yield of recombinant polypeptides give them certain advantages in the research and development of new drugs.

### 4.2 Analgesic activity

Studies have shown that using the whole body or tails of *Scorpio* in the treatment of somatic pain, visceral pain, neuralgia, and cancerous pain yields good therapeutic effects (Liu and Ma, 1993). The hot plate tail-flick test and acetic acid-induced writhing test were used to detect the analgesic effects of scorpion bodies and tails, respectively, and the results indicated that both exhibit significant analgesic effects (Liu and Ma, 1993). *Scorpio* can exert an analgesic effect on rats with bone cancer pain by inhibiting bone destruction and suppressing the activation of spinal astrocytes and microglia (Yu et al., 2020).Since analgesia is the main pharmacological effect of *Scorpio*, in recent years, many scholars have been committed to isolating and purifying single-component analgesic active peptides from scorpion venom, the main active component of *Scorpio*. The comparison between the peptides with analgesic activity and the positive control drug is summarized in Table 7.

**TABLE 7:** Peptides with analgesic activity from *B*. *martensii scorpions*.

Name	Model	Dose- inhibition efficiency		Reference
		Peptide	Positive control	
Bmk AEP	Acetic acid-induced writhing pain model	0.75 mg/kg-50%	0.2 mg/kg-70.7%	Cao (2004)
BmKBTx	Acetic acid-induced writhing pain model	Slightly less analgesic than morphine		Cui et al. (2017)
BmNaL-3SS2	Acetic acid-induced writhing pain model	Exhibit greater analgesic effects than morphine		Cui et al. (2017)
BmK AGAP	Acetic acid-induced writhing pain model and hot plate test	1.0 mg/kg-60%	1.5 mg/kg-35%	Liu et al. (2003)
BmK AGP-SYPU1	Acetic acid-induced writhing pain model	1.0 mg/kg-68.8%	1.0 mg/kg-65.2%	Yu et al. (2011)
BmK AGP-SYPU2	Acetic acid-induced writhing pain model and hot plate test	1.42 mg/kg-50%	1.5 mg/kg--	Shao et al. (2014)
BmK IT-AP	Acetic acid-induced writhing pain model	1 mg/mL-54.4%	80 mg/mL- 62.4%	Xiong et al. (1999)
BmK IT1	Acetic acid-induced writhing pain model	0.7 mg/kg-50%	0.2 mg/kg-70.7%	Cao (2004)
BmK AngP1	Acetic acid-induced writhing pain model	5 μg/g-43.0%	80 μg/g-62.4%	Guan et al. (2001b)
BmK dITAP3	Acetic acid-induced writhing pain model	5 μg/g-44.8%	-	Guan et al. (2001a)
BmK Ang M1	Acetic acid-induced writhing pain model	0.39 mg/kg-50%	0.25 mg/kg-78.3%	Cao (2004)
BmK AS	Acetic acid-induced writhing pain model and hot plate test	1.42 mg/kg-50%	2.0 mg/kg-80%	Shao et al. (2007b)

Voltage-gated sodium channels (VGSCs) have been identified as crucial components in the molecular mechanisms underlying pain signaling pathways. Among the various VGSC subtypes, Nav1.3, Nav1.7, and Nav1.8 have been specifically implicated in nociceptive processing and pain pathogenesis (Li et al., 2019). It has been demonstrated that blocking these sodium channels significantly attenuates or completely abolishes pain responses. BmNaL-3SS2 and BmKBTx, two additional toxins identified from the cDNA library of *B*. *martensii* venom glands, have been shown to exhibit analgesic activity through selective inhibition of Nav1.7 channels (Cui et al., 2017). BmK AS, a *β*-toxin, was shown to dose-dependently inhibit sodium currents without affecting potassium currents or Ca^2+^ influx, indicating its specific targeting of VGSCs(Liu et al., 2008). In contrast to the aforementioned sodium channel-targeting peptides, BmK-YA specifically interacts with opioid receptors, demonstrating binding affinity for μ-, δ-, and κ-opioid receptor subtypes (Yan et al., 2012). Notably, BmK-YA exhibits a 72-fold lower EC_50_ value at μ-opioid receptors compared to morphine, suggesting a potentially reduced risk of receptor-mediated adverse effects (Yan et al., 2012).

At present, the commonly used analgesic drugs in clinical practice are opioid analgesics and non-steroidal anti-inflammatory drugs, which often lead to dependence and addiction. Active components from *Scorpio* and scorpion venom have strong inhibitory effects on various acute and chronic pains, with most exhibiting greater efficacy than morphine, and they are non-addictive. With further development of the analgesic components in scorpion venom, it is expected that new analgesic drugs with non-addictive properties, long-lasting effects, and high safety will be developed and widely used in clinical practice in the future, benefiting patients.

### 4.3 Anti-epilepsy activity

Accumulating evidence has demonstrated that venom-derived peptides from *B*. *martensii* exhibit high specificity toward VGSCs, positioning them as promising candidates for anti-epileptic therapeutics (Xiao et al., 2022). In preclinical studies, the anti-epileptic peptide AEP (28 mg/kg) significantly suppressed seizures induced by coriolide lactone and cephaloridine in rat models, and its efficacy outperformed that of diazepam. Meanwhile, AEP showed no adverse effects on cardiovascular parameters such as heart rate, blood pressure, or electrocardiographic profiles (Zhou et al., 1989). Further investigation into the anticonvulsant properties of BmK IT2 revealed its therapeutic potential in a pilocarpine-induced status epilepticus (SE) model. Administration of BmK IT2 markedly prolonged the latency to SE onset, attenuated SE severity, and suppressed hippocampal c-Fos expression during epileptogenesis, suggesting its role in modulating neuronal hyperexcitability (Zhao et al., 2008). Notably, BmK AS, a peptide targeting receptor site 4 of VGSCs, demonstrated potent inhibition of pentylenetetrazole (PTZ)-induced seizures (Zhao et al., 2011a). This selective interaction highlights BmK AS as a novel molecular probe for elucidating the pathophysiological role of specific sodium channel subtypes in epilepsy. Additionally, this interaction underscores BmK AS’s translational value in developing subtype-selective antiepileptic drugs.

### 4.4 Antibiosis activity

*Scorpio* exhibits broad-spectrum antibacterial activity and exerts a certain inhibitory effect on both Gram-positive and Gram-negative bacteria, primarily mediated by bioactive components including peptides, steroids, and quinones. Peptides with antibacterial activity are summarized in Table 8, including BmK bpp, BmKn2, and BmK AS. Mechanistic studies reveal that BmKn2 rapidly lyses *Staphylococcus aureus* by binding to lipoteichoic acid (LTA) on the bacterial surface, disrupting membrane integrity, and degrading the cell wall (Cao et al., 2012).

**TABLE 8:** Peptides with antibacterial activity from *B. martensii scorpions*.

Name	Bacteria	Minimum inhibitory concentrations (mics)	Reference
BmK bpp	Gram-negative bacteria	2.3–68.2 μM	Zeng et al. (2012)
	*Bacillus subtilis, Listeria monocytogenes, Micrococcus luteus, Enterococcus faecalis*	24.4 μM, 5.7 μM, 20.3 μM, 57.1 μM	
BmKn2	penicillin-resistant *Staphylococcus aureus* (PRSA), methicillin-resistant *S. aureus* (MRSA)	0.6–50 μg/mL	Cao et al. (2012)
BmK AS	*S. aureus, Salmonella typhi, Escherichia coli, Pseudomonas aeruginosa*	4.5–10 μM	Ji et al. (1999), Shao et al. (2008)

Furthermore, cardiac glycosides derived from scorpion venom exhibit notable antimicrobial properties. A structurally unique cardiac glycoside, identified as 3*β*-acetoxy-2,14,22-trihydroxy-19-hydroxymethyl-9*α*,5*β*,14*β*-card-20 (22)-enolide, demonstrated significant inhibitory activity against the Gram-positive bacterium *Bacillus subtilis*, with a MIC of 15 μg/mL (Gao et al., 2014). These findings underscore the diversity of antimicrobial components in scorpion venom and their potential as novel therapeutic agents against drug-resistant infections.

### 4.5 Anticoagulant and antithrombotic activity

Recent studies have demonstrated that scorpion venom peptides exhibit potent anticoagulant and antithrombotic activities. In murine thrombosis models and zebrafish thrombosis assays, peptides such as BmK-M10 and BmKITc showed significant antithrombotic effects, with BmKITc exhibiting superior efficacy in a concentration-dependent manner (Sun et al., 2021). *In vitro* platelet aggregation assays revealed that scorpion venom peptides (SVPs) significantly inhibited thrombin-induced platelet aggregation in rabbits in a dose-dependent manner (Lv et al., 1995). Further investigations into the effects of scorpion venom anticoagulant peptides (SVAPs) on hemorheology demonstrated that SVAPs reduced plasma viscosity (PV), fibrinogen (Fib) levels, and erythrocyte aggregation while enhancing erythrocyte deformability. These findings suggest that SVAPs improve blood fluidity by lowering whole blood viscosity (BV), which may contribute to their antithrombotic and fibrinolytic mechanisms (Song et al., 2002). Additionally, scorpion-derived bioactive peptides have been shown to enhance mesenteric microcirculation in rats by increasing capillary blood flow and vascular diameter, thereby preventing thrombus formation (Tian and Zheng, 2004). Mechanistic studies indicate that scorpion venom fibrinolytic peptides upregulate prostacyclin (6-keto-PGF1*α*) and nitric oxide (NO) levels while modulating the activity of plasminogen activator inhibitor-1 (PAI-1) and tissue plasminogen activator (tPA). These effects protect endothelial cells under hypoxic conditions and enhance their anticoagulant and fibrinolytic functions (Wang and Wu, 2010). Furthermore, scorpion venom peptides stimulate NO secretion in endothelial cells, leading to elevated intracellular cyclic guanosine monophosphate (cGMP) levels, which inhibit platelet aggregation. Concurrently, they suppress thrombin-induced increases in intracellular calcium concentration, further attenuating prothrombotic pathways (Tian and Zheng, 2004).

### 4.6 Antiviral activity

According to the Chinese Pharmacopoeia, *Scorpio* is traditionally described as possessing therapeutic properties to “neutralize toxins and dissipate pathogenic accumulations.” Modern pharmacological studies have further revealed its broad-spectrum antiviral activity against the influenza virus, dengue virus (DENV), hepatitis C virus (HCV), herpes simplex virus (HSV), and human immunodeficiency virus (HIV) (Andrea et al., 2022). Notably, the scorpion venom-derived peptide HP-1090 exhibits potent anti-HCV effects by disrupting the viral capsid structure and suppressing genomic replication, with a half-maximal inhibitory concentration (IC_50_) of 7.62 μg/mL (Yan et al., 2011). Another antiviral peptide, AVP, targets the HIV-1 envelope glycoprotein gp120, competitively inhibiting its interaction with the CD4 receptor to block viral entry into host cells (da Mata et al., 2017). Beyond direct viral neutralization, AVP modulates host immune responses, potentially interfering with multiple stages of the viral life cycle (Andrea et al., 2022). Additionally, aqueous extracts from lyophilized scorpion venom demonstrate dose-dependent inhibition of HSV, respiratory syncytial virus (RSV), and enterovirus 71 (EV71), with optimal inhibitory concentrations of 2 mg/mL, 20 μg/mL, and 2 mg/mL, respectively (Zhang et al., 2019).

### 4.7 Therapeutic activity of cardiovascular diseases

Martentoxin, a 37-amino-acid peptide belonging to the *α*-KTx (alpha-potassium channel toxin) toxin family, is purified from the venom of *B*. *martensii*. This neuroactive peptide significantly attenuates tumor necrosis factor-*α* (TNF-*α*)-induced NO overproduction through dual mechanisms: inhibition of iNOS (inducible nitric oxide synthase) activity and blockade of calcium-activated potassium channels (Busse et al., 1991; Shi et al., 2008; Wang et al., 2013). Pathological NO overproduction is critically involved in multiple disease processes, including gram-negative bacterial sepsis, cardiac dysfunction, and ischemia-reperfusion injury in cerebral and cardiovascular systems (Silvia Llorens, 2003). These findings establish Martentoxin as a potential therapeutic agent for managing NO-mediated pathological conditions. Bumarsin, a 72-amino-acid polypeptide isolated from BmK venom, demonstrates potent inhibition of 3-hydroxy-3-methylglutaryl-coenzyme A (HMG-CoA) reductase, achieving 32% suppression of enzyme activity at a concentration of 0.6 mM(Chai et al., 2012). HMG-CoA reductase inhibitors are clinically established for managing hypercholesterolemia, a major risk factor for atherosclerosis and associated cardiovascular disorders. Notably, Bumarsin exhibits superior pharmacological efficacy compared to simvastatin, a first-line statin drug. While simvastatin achieves only 35% HMG-CoA reductase inhibition at 10 mM concentration, Bumarsin demonstrates comparable inhibition (32%) at 60-fold lower concentration (0.6 mM) (Chai et al., 2012). Moreover, Bumarsin modulates cholesterol homeostasis through upregulation of key regulatory proteins, including apolipoproteins (Apo-A1, Apo-E, Apo-CI/II/III) and the steroidogenic acute regulatory protein (StAR) (Chai et al., 2012). These findings collectively highlight *Scorpio*’s multifactorial mechanism of action and its clinical potential as a novel therapeutic agent for hypercholesterolemia management.

### 4.8 Other activities

Alzheimer’s disease (AD), a neurodegenerative disorder characterized by progressive cognitive decline, is closely associated with acetylcholine deficiency. Acetylcholinesterase (AchE) and butyrylcholinesterase (BchE), key enzymes catalyzing acetylcholine hydrolysis, represent critical therapeutic targets (Zhang et al., 2022a). Guanidine alkaloids (martensine A/B) isolated from *Scorpio* exhibit dual inhibition of AchE and BchE, potentially through interactions with catalytic and peripheral anion sites. These alkaloids also demonstrate bio metal chelation capacity (Cu^2+^, Fe^2+^, Zn^2+^, Al^3+^), suggesting multi-target therapeutic potential for AD (Liu et al., 2021b). Furthermore, a novel *β*-carbonyl saccharide alkaloid (Harmonyl-*β*-D-glucopyranoside) from *Scorpio* non-competitively inhibits *α*-glucosidase (IC_50_ = 24 μM), showing promise for metabolic disorder management (Kim, 2013). In cancer therapeutics, polypeptide PESV synergizes with driamycin to reverse multidrug resistance in leukemia K562/A02 stem cells by downregulating P-gp, BCRP, MDR1, and PI3K/NF-κB pathway components (Yang et al., 2016). Additionally, the neuroprotective scorpion venom heat-resistant peptide (SVHRP) exhibits neuroprotective effects by reducing neurological deficit scores, inhibiting edema formation, decreasing infarct volume and neuronal loss, and protecting primary neurons against oxygen-glucose deprivation/reoxygenation (OGD/R)-induced injury (Wang et al., 2020).

In summary, *Scorpio* exerts a wide range of pharmacological effects, and research on the material basis for exerting these effects focuses primarily on the active peptides of scorpion toxins. However, it must be clearly stated that the vast majority of these findings are based on *in vitro* models and animal experiments, lacking validation from human clinical trials. This limitation hinders our progress in translating these promising active substances into clinical drugs. BmK AGAP, a potential candidate molecule, has demonstrated heterologous expression and maintained its pharmacological effects while increasing its yield. In the future, it may be employed as a monotherapy drug to systematically promote the pre-clinical and clinical development that meets the standards. In light of the prevalent challenges associated with peptide drugs, including their limited membrane permeability and instability *in vivo*, there is an imperative to explore the potential of nanotechnology, such as liposomes and polymer nanoparticles, for the encapsulation of these peptides. This approach aims to enhance their bioavailability, target specificity, and controlled release, thereby facilitating their clinical success.

## 5 Toxicity and side effects

Research has demonstrated that although *Scorpio* exhibits significant pharmacological efficacy, its use at high doses may induce multiple adverse reactions. When investigating the analgesic effects of Centipede-Scorpion Powder in mice, the serum levels of alanine aminotransferase (ALT) and aspartate aminotransferase (AST) in the low- and medium-dose groups (20 and 50 mg/kg) showed no significant differences compared to the control group. However, in the high-dose group (80 mg/kg), both ALT and AST levels were significantly elevated, indicating increased permeability of hepatocyte membranes and suggesting mild hepatic injury at this dosage (Xu et al., 2018). Modern pharmacological studies have confirmed that the toxic components of *Scorpio* primarily originate from its venom, which can readily induce multi-system toxicity including neurotoxicity, cardiovascular damage, urinary system injury, and respiratory depression (Ma, 2019). The toxicological effects of scorpion venom predominantly stem from their interaction with VGSCs. BmK NT1 enhances sodium influx in cerebellar granule cells (CGCs) and inhibits the fast inactivation of VGSCs, mimicking the pharmacological behavior of *α*-scorpion toxins. Neurotoxicity induced by *α*-scorpion toxins via VGSC activation is attributed to intracellular Ca^2+^ overload mediated through NMDA receptor-dependent pathways and Na^+^/Ca^2+^ exchanger mechanisms (He et al., 2017). Notably, BmK M7, an *α*-scorpion toxin, exerts cardiotoxic effects by binding to cardiac Na^+^ channels and altering their electrophysiological properties (Guan et al., 2002). BmK I, another *α*-scorpion toxin comprising 64 amino acid residues, has been identified as specifically binding to receptor Site-3 and prolong the inactivation phase of VGSCs(Possani et al., 2010). Intrahippocampal administration of BmK I elevated c-Fos expression and caused significant morphological changes in the hippocampus, leading to neuronal loss in distinct hippocampal subregions and inducing convulsive behaviors in rats (Bai et al., 2006). It is important to note that quantitative toxicokinetic parameters (such as LD_50_ (median lethal dose) and therapeutic index) are lacking for most isolated scorpion venom components. Establishing quantitative thresholds for ‘safe’ exposure levels of these toxic constituents is therefore essential for ensuring clinical safety.

*Scorpio* undergoes multiple preparatory methods—such as boiling (in plain water or saltwater), mint processing, licorice rinsing, and alcohol rinsing—prior to clinical application, aimed at mitigating toxicity while preserving pharmacological activity (Zhang et al., 2024). Adjuvants are also used to achieve the same goal. For example, Radix Glycyrrhizae and honey, as sweet-natured harmonizing agents, mitigate the violent toxicity of scorpion venom; mint (dispelling wind and detoxifying) and glutinous rice (moistening dryness) enhance its wind-dispelling and analgesic effects; and vinegar processing simultaneously masks odors, neutralizes toxins, and directs the medicinal properties toward the liver channel via its sour nature, thereby doubling the efficacy of vinegar-processed scorpion in resolving masses, freeing collateral channels, and alleviating pain (Ma, 2019; Zhang et al., 2024). Because chitosan has a good adsorption performance for trace heavy metal ions such as lead, the subsequent processing of scorpions with chitosan significantly lowers heavy metal content in processed scorpions (Xiang, 2014). Overall, proper processing methods significantly reduce toxicity, while maximising the retention of pharmacological activity. However, research on the mechanisms underlying this detoxification remains limited, and the patterns of component changes and toxicity variations before and after processing are not yet fully understood. Future studies could integrate the analysis of *Scorpio*’s active components with its toxicity, employing correlation analysis to elucidate the scientific basis for detoxification through processing.

## 6 Conclusion and future perspectives

This study reviews the core research advances on the Chinese medicinal scorpion (*B*. *martensii*) based on extant literature. Traditional applications trace back to its first documented medicinal value in the *Shu Ben Cao*, with the *Kaibao Bencao* explicitly noting its use in treating spasms and convulsions. As demonstrated in the Qing Dynasty summaries, the efficacy of this treatment in dispersing blood heat and expelling wind-dampness is well-established, constituting a significant contribution to the long-standing and systematic medicinal history. The chemical constituents of the venom are centered on scorpion venom peptides as the core active substances, supplemented by steroidal derivatives, alkaloids, amino acids, and other components, collectively forming the material basis for its pharmacological activity. The pharmacological effects span anticancer, analgesic, antiepileptic, antibacterial, anticoagulant, and antithrombotic activities. Scorpion venom peptides (e.g., BmK AGAP, PESV) have been shown to possess significant therapeutic potential through mechanisms such as ion channel regulation and apoptosis induction. However, it should be noted that high-dose administration of *Scorpio* and peptides may induce adverse reactions, including liver damage and neurotoxicity. Whilst processing methodologies such as boiling and licorice rinsing have been demonstrated to reduce toxicity, the underlying mechanisms remain unclear.

With a history of use in traditional Chinese medicine for over a millennium, *B*. *martensii* has been studied for its potential pharmacological applications, though further research is needed to validate its therapeutic efficacy. Firstly, there is a paucity of clinical evidence, as all pharmacological activities are based solely on *in vitro* or animal experiments, lacking human clinical trial data. Secondly, key mechanisms remain unelucidated, with no molecular mechanisms for processing and detoxification identified and no established quality control standards. Thirdly, there is a disconnect between component research and clinical application. Clinical use relies on processed scorpions, which are ground into powder and mixed with other medicinal materials before being administered orally to patients. However, modern reductionist studies focus on isolated scorpion venom components, without thoroughly exploring the significant differences between the effects of pure peptides and those of processed crude medicinal materials.

In the field of Traditional Chinese Medicine, future research on *Scorpio* should prioritize “clinical translation” as the central focus. This approach aims to address the discrepancy between “modern studies focusing on isolated scorpion venom components” and “clinically used processed scorpions.” To this end, researchers should employ interdisciplinary technologies to overcome existing limitations, thereby facilitating progress in this area of study. Omics technologies need to advance from the current preliminary exploration of scorpion venom components using transcriptomics and proteomics to an integrated “transcriptomics-proteomics-metabolomics” analysis (Gao et al., 2021). This not only involves excavating toxin genes specifically expressed in scorpion venom gland cells to optimize the expression of recombinant peptides, but also requires analyzing the interactions between scorpion venom components and other constituents (such as steroids and alkaloids). It is essential to clarify the differential mechanisms of component synergy and pharmacodynamic effects between pure peptides and crude, processed medicines. In the field of structural biology, it is imperative to elucidate the intricate structures of scorpion venom peptides bound to their targets (e.g., Nav1.7, μ-opioid receptors) (Yan et al., 2012; Cui et al., 2017). This provides a foundation for peptide modification (e.g., reducing the cardiotoxicity of BmK M7) (Guan et al., 2002). Concurrently, research should be conducted on the target network involved in the multi-component co-action, in conjunction with the compositional characteristics of processed scorpions. In addition to enhancing the druggability of scorpion venom peptides, contemporary drug delivery systems must be engineered to preserve the synergistic advantages of multiple components when crude, processed medicines are employed (Yang et al., 2024).

Although this review summarizes a large amount of research on *Scorpio*, there are some limitations. Firstly, our literature search was limited to English and Chinese databases (PubMed, CNKI, etc.), and we acknowledged that this may have excluded relevant studies published in other languages or regional databases—potentially narrowing the scope of evidence synthesized. Secondly, we implemented a pragmatic quality filter during literature selection—excluding studies with unclear experimental methods, insufficient sample sizes, or unvalidated outcome measures, and most of the included studies reported positive outcomes, which may reflect publication bias. Thirdly, this review did not fully incorporate international guidelines relevant to processed *B*. *martensii* research, which may limit the international applicability of its findings.

## Glossary

AChE: Acetylcholinesterase
AD: Alzheimer’s disease
ALT: Alanine aminotransferase
AST: Aspartate aminotransferase
BChE: Butyrylcholinesterase
bFGF: Basic fibroblast growth factor
*B*. *martensii*: *Buthus martensii* Karsch
BV: Blood viscosity
CGCs: Cerebellar granule cells
cGMP: Cyclic guanosine monophosphate
CNKI: China national knowledge infrastructure
DBPs: Disulfide-bridged peptides
DENV: Dengue virus
DNA: Deoxyribonucleic acid
EGFR: Epidermal growth factor receptor
EMT: Epithelial-mesenchymal transition
EV71: Enterovirus 71
FN: Fibronectin
GC-MS: Gas chromatography-mass spectrometry
HCV: Hepatitis C virus
HIV: Human immunodeficiency virus
HMG-CoA: Hydroxy methylglutaryl coenzyme A
HS-SPME: Headspace solid-phase microextraction
HSV: Herpes simplex virus
IC_50_: Half maximal inhibitory concentration
LTA: Lipoteichoic acid
MAPK: Mitogen-activated protein kinase
MICs: Minimum inhibitory concentrations
MMPs: Matrix metalloproteinase
MRSA: Methicillin-resistant *Staphylococcus aureus*
NDBPs: Non-disulfide-bridged peptides
NF-*κ*B: Nuclear factor kappa-B
NK: Natural killer
NO: Nitric oxide
PAI-1: Plasminogen activator inhibitor-1
PESV: Polypeptide extract from scorpion venom
PI3K: Phosphoinositide 3-kinase
PRSA: Penicillin-resistant *Staphylococcus aureus*
PTZ: Pentylenetetrazole
PV: Plasma viscosity
RSV: Respiratory syncytial virus
SVPs: Scorpion venom peptides
TNF-*α*: Tumor necrosis factor *α*
tPA: Tissue plasminogen activator
uPA: Urokinase-type plasminogen activator
VEGF: Vascular endothelial growth factor
VGSCs: Voltage-gated sodium channels
